# Improved Isolation and Culture of Urine-Derived Stem Cells (USCs) and Enhanced Production of Immune Cells from the USC-Derived Induced Pluripotent Stem Cells

**DOI:** 10.3390/jcm9030827

**Published:** 2020-03-18

**Authors:** Kyeongseok Kim, Minchan Gil, Ahmed Abdal Dayem, Sangbaek Choi, Geun-Ho Kang, Gwang-Mo Yang, Sungha Cho, Yeojin Jeong, Se Jong Kim, Jaekwon Seok, Hee Jeong Kwak, Subbroto Kumar Saha, Aram Kim, Ssang-Goo Cho

**Affiliations:** 1 Department of Stem Cell & Regenerative Biotechnology and Incurable Disease Animal Model and Stem Cell Institute (IDASI), Konkuk University, 120 Neungdong-ro, Gwangjin-gu, Seoul 05029, Korea; proproggs@naver.com (K.K.); minchangil@gmail.com (M.G.); ahmed_morsy86@yahoo.com (A.A.D.); hjyone@naver.com (S.C.); geunhokang@naver.com (G.-H.K.); slayersgod@nate.com (G.-M.Y.); shgool@naver.com (S.C.); jyj05787@gmail.com (Y.J.); rlatpwhdc@nate.com (S.J.K.); tjrwornjs@naver.com (J.S.); h_jeong9581@naver.com (H.J.K.); subbroto1986@gmail.com (S.K.S.); 2 Department of Urology, Konkuk University Medical Center, Konkuk University School of Medicine, Seoul 05029, Korea; arkim@kuh.ac.kr

**Keywords:** urine stem cell, Y-27632, matrigel, 3,2′-DHF, 3,4′-DHF, hematopoietic stem cell, kidney organoid, cell isolation, hiPSC

## Abstract

The availability of autologous adult stem cells is one of the essential prerequisites for human stem cell therapy. Urine-derived stem cells (USCs) are considered as desirable cell sources for cell therapy because donor-specific USCs are easily and non-invasively obtained from urine. Efficient isolation, expansion, and differentiation methods of USCs are necessary to increase their availability. Here, we developed a method for efficient isolation and expansion of USCs using Matrigel, and the rho-associated protein kinase (ROCK) inhibitor, Y-27632. The prepared USCs showed significantly enhanced migration, colony forming capacity, and differentiation into osteogenic or chondrogenic lineage. The USCs were successfully reprogramed into induced pluripotent stem cells (USC-iPSCs) and further differentiated into kidney organoid and hematopoietic progenitor cells (HPCs). Using flavonoid molecules, the isolation efficiency of USCs and the production of HPCs from the USC-iPSCs was increased. Taken together, we present an improved isolation method of USCs utilizing Matrigel, a ROCK inhibitor and flavonoids, and enhanced differentiation of USC-iPSC to HPC by flavonoids. These novel findings could significantly enhance the use of USCs and USC-iPSCs for stem cell research and further application in regenerative stem cell-based therapies.

## 1. Introduction

Stem cells have been investigated extensively for their potential medical use in regenerative medicine [1,2,3]. Stem cells can be expanded and differentiated into specific target cells for replacing diseased or damaged tissues. Therefore, stem cells have been studied for cell therapies for diseases including spinal cord injuries [4], type 1 diabetes [5], Parkinson’s disease [6], Alzheimer’s disease [7], amyotrophic lateral sclerosis [8], burns [9], heart disease [10], stroke [11], cancer [12], and osteoarthritis [13]. However, obtaining enough numbers of appropriate stem cells has been challenging because of difficulties in isolating stem cells and ethical issues in using pluripotent embryotic stem cells [14,15,16]. Donor-specific autologous adult stem cells such as adipose- and bone marrow-derived stem cells are considered as appropriate cells for cell therapy because of the absence of complete immune rejection response and minimal ethical issues [17]. Adipose-derived stem cells (ADSCs) can be easily obtained through liposuction. However, the skin incision procedure is traumatic and invasive causing cosmetic issues and rarely medical complications [18,19,20]. Invasive extraction procedure from bone marrow is also needed to obtain bone marrow-derived stem cells (BMSCs) [16,21]. Therefore, a safer and easier way of autologous adult stem cell isolation is desirable.

Urine-derived stem cells (USCs) are subpopulations of cells isolated from the urine, which share similar biological properties with other adult mesenchymal stem cells (MSCs) such as ADSCs and BMSCs [22]. USCs originate from the kidney renal tubules or papilla [23]. USCs can be obtained daily over the entire lifetime of the patient in a non-invasive manner without health risks and can be expanded to large numbers from a single clone. These cells have higher telomeric activity and longer telomeres than BMSCs and, therefore, have a very high proliferative capacity [24,25]. The number of obtained cells from previously established protocols could be considered enough for the use of regenerative medicine in some applications [23,25]. However, improving the yield of USC isolation is inevitably necessary for its potential use in further laboratory and clinical applications. In this study, Matrigel, Y-27632, and flavonoids were examined for their ability to enhance isolation and subsequent expansion and differentiation of USCs.

Matrigel is extracted from Engelbreth–Holm–Swarm mouse tumors and contains laminin, collagen, entactin, heparin sulfate proteoglycan, and growth factors [26]. Matrigel has been used extensively in feeder-free 2D or 3D cultures of human pluripotent stem cells (hPSCs) to support cell adhesion and survival and maintain them in an undifferentiated state of growth [27,28]. Y-27632 is an inhibitor of the rho-associated protein kinase (ROCK) signaling pathway [29]. It has been shown to prevent dissociation-induced apoptosis of hiPSCs and to augment survival of cryopreserved cells [30]. Furthermore, Y-27632 has been shown to promote differentiation of hBMSCs into keratinocyte-like cells [31] and to increase the post-thaw viability of cryopreserved hBMSCs [32].

Flavonoids are natural compounds that are abundant in plant pigments and a wide range of foods [33,34]. We have shown that flavonoids have a wide range of activities, such as anti-apoptotic [35,36,37], anti-viral [38,39], and anti-diabetic functions [40], adipogenesis modulation ability [21], pluripotency marker expression ability, and neuroprotective properties [41].

In this study, we isolated USCs from six different donors and characterized their gene expression profiles. We also identified the promoting effect of Matrigel, Y-27632, and 3,2′-dihydroxyflavone (3,2′-DHF) and 3,4′-dihydroxyflavone (3,4′-DHF) flavonoids on USC isolation yield and properties. Human induced PSC (iPSC) technology also holds great potential for personalized regenerative medicine, drug discovery, and disease modeling without concerns for ethical issues like those associated with human embryonic stem cells [42]. USCs were reprogrammed to iPSCs. The gene expression profile of USC-derived iPSCs (USC-iPSCs) and the differentiation capacity to kidney organoids and hematopoietic progenitor cells (HPCs) were investigated. Overall, we characterized the USCs and USC-iPSCs and suggest treatments for improved preparation and isolation of USCs for laboratory and clinical applications.

## 2. Materials and Methods

### 2.1. Cells

All human cells were obtained and managed with the approval of the institutional review board (IRB) of the Konkuk University Bioethics Committee. Human dermal fibroblasts (HDFs) with passage 5, adipose-derived stem cells (ADSC) with passage 1, and Wharton’s jelly-derived MSCs (WJ-MSCs) with passage 2 were obtained from Cell2in (Seoul, Republic of Korea) (IRB 7001355-201507-BR-181). The human peripheral blood mononuclear cells (PBMCs) were prepared from the blood of healthy donors using density gradient centrifugation via Ficoll-Paque PLUS (GE Healthcare, Chicago, IL, USA) (IRB 7001355-201507-BR-072) as we described [43]. Basically, the USCs isolation from human urine samples were carried out based on the previously established protocol [44]. For the isolation of USCs, urine was collected from six donors aged 15 to 50 years (IRB 7001355-201507-BR-072). The purpose of the isolation and all the procedures were described to the donor, and subsequently their consent was obtained. Approximately 100–200 mL urines were collected from the donors and centrifuged for 10 min at 400 g. After that, cell pellets were washed once with phosphate-buffered saline (PBS) supplemented with Antibiotics-Antimycotics (Thermo Fisher Scientific, Waltham, MA, USA). Cells were then plated in primary medium containing 0.5 mL Dulbecco’s Modified Eagle’s Medium (DMEM)/Ham’s Nutrient Mixture F12 (Thermo Fisher Scientific) consisting of 10% FBS, 1% penicillin/streptomycin and 0.5 mL urine renal epithelial growth medium (REGM, Basel, Switzerland) supplemented with or without Y-27632 (STEMCELL Technologies, Vancouver, Canada) on gelatin- or Matrigel (BD Biosciences, Franklin lake, NJ, USA)-coated 6-well culture plates. USCs were expanded in proliferation medium containing 50% DMEM high glucose consisting of 10% FBS, 1% penicillin/streptomycin and 50% REGM under standard culture conditions (37 °C, 5% CO_2_). For passaging the USCs, cells were washed once with PBS and gently dissociated to single cells with 0.25% Trypsin/EDTA (Thermo Fisher Scientific) and seeded again onto cell culture dishes. For cryopreservation of USCs, cells were detached using 0.25% Trypsin/EDTA. 5 × 10^5^ of detached cells were mixed with freezing medium (FBS 60%, REGM medium 30%, 10% Dimethyl Sulfoxide (DMSO, Santa Cruz Biotechnology, Dalla, TX, USA)) in cryogenic vials (Corning, Corning, NY, USA).

The flavonoids 3-hydroxyflavone (3-HF), 3,2′-DHF, 3,3′-dihydroxyflavone (3,3′-DHF), or 3,4′-DHF were purchased from the INDOFINE Chemical Company (Hillsborough, NJ, USA) and treated when urine cells were initially plated on gelatin coated plate to examine the effect of flavonoids on the isolation of USCs.

### 2.2. Flow Cytometry (FACS) Analysis

For FACS, cells were dissociated using 0.25% Trypsin/EDTA, washed with PBS, resuspended in blocking buffer consisting of 0.5% BSA and 2% FBS in PBS and kept for 30 min at 4 °C. Then, 1 × 10^6^ cells were incubated with antibodies against the human antigens CD34 (1:100, Santa Cruz Biotechnology), CD45 (1:100, Santa Cruz Biotechnology), CD73 (1:100, Santa Cruz Biotechnology), CD90 (1:100, Santa Cruz Biotechnology), and CD105 (1:100, Santa Cruz Biotechnology) for 1 h at 4 °C. A nonspecific isotype-matched antibody was used as a control. Afterwards, cells were incubated with a fluorescent dye conjugated secondary antibody (Alexa Fluor 488-conjugated donkey anti-rabbit IgG (H+L) (1:500, Invitrogen, Carlsbad, CA, USA) or Alexa Fluor 546-conjugated rabbit anti-mouse IgG (H+L) (1:500, Invitrogen)) and diluted in FACS buffer (0.5% BSA and 0.05% sodium azide in 1× PBS) per the manufacturer’s recommendations in the dark for 30 min. After washing with FACS buffer, the cells were fixed using 4% paraformaldehyde buffer (Sigma, St. Louis, MI, USA) for 30 min and then analyzed using a flow cytometer (FACSCalibur Flow Cytometer, BD Biosciences).

### 2.3. RNA Isolation and Reverse Transcriptase- Polymerase Chain Reaction (RT-PCR)

Total cellular RNA was isolated using Trizol reagent (Thermo Fisher Scientific) according to the manufacturer’s protocol. For cDNA synthesis, 2 μg of total RNA was used in a 25 μL reaction containing 200 U of M-MLV reverse transcriptase (Promega, Madison, WI, USA), 10 mM dNTP, and 20 pmol Oligo (dT). For PCR, 1 μL cDNA template was mixed with 10 pmol forward and reverse primers each and 4 μL rTaq Plus 5 × PCR Master Mix (ELPIS-BIOTECH, Daejeon, Republic of Korea), the volume was then made up to 20 µL using H_2_O. The PCR was performed using the 9902 Veriti Thermal Cycler (Applied Biosystems, Waltham, MA, USA) under the following conditions: initial denaturing at 95 °C for 3 min and then 35 cycles of 10 s at 95 °C, 10 s at appropriate annealing temperature and 10 s at 72 °C, followed by 5 min at 72 °C. The PCR was performed in triplicate. The PCR products were visualized in a 1.5% agarose gel stained with ethidium bromide. Images of the resulting gels were captured under ultra-violet light using Powershot A520 (Canon, Tokyo, Japan). Sequences of the primers used are given in Supplementary Table S1.

### 2.4. RNA Sequencing (RNA-seq) and Gene Ontology (GO)

RNA-seq was performed at BMS Korea Corporation (Gimpo, Republic of Korea). Briefly, the quantity and quality of the isolated total RNA were evaluated using the Agilent 2100 bioanalyzer RNA kit (Agilent Technologies, Santa Clara, CA, USA) and processed for preparing the mRNA sequencing library using the Illumina TruSeq Stranded mRNA Sample Preparation kit (Illumina, San Diego, CA, USA) according to the manufacturer’s protocol. The quality and size of libraries were assessed using the Agilent 2100 bioanalyzer DNA kit (Agilent). All libraries were quantified by qPCR using CFX96 Real Time System (Biorad Laboratories, Hercules, CA, USA) and sequenced on the NextSeq500 sequencers (Illumina) with a paired-end 75 bp plus single 8 bp index read run. To quantify the mapped reads on the reference genome into the gene expression values, Cufflinks [45] with the strand-specific library option and other default options were used. The gene annotation of the reference genome hg19 from the UCSC genome (https://genome.ucsc.edu) in GTF format was used as a gene model. The expression values were calculated in fragments per kilobase of transcript per million fragments mapped (FPKM). The differentially expressed genes (DEGs) between the two selected biological conditions were analyzed by Cuffdiff software in Cufflinks package (Trapnell Lab, Seattle, WA, USA) [46] with the strand-specific library option, and other default options. To compare the expression profiles among the samples, the normalized expression values of the selected few hundred DEGs were clustered unsupervisedly by in-house R scripts. In order to obtain GO term annotation results, the genes classified in RNA-Seq from g:Profiler were analyzed (https://biit.cs.ut.ee/gprofiler/gost).

### 2.5. Cell Proliferation Assay

For the cell proliferation assay, 2 × 10^4^ USCs from donor 1 with passages between 1 and 3 were seeded per well in 12-well culture plates in 1 mL of USC proliferation medium and incubated at 37 °C. USCs were enumerated using a hemocytometer with trypan blue solution (Biorad Laboratories) at 24, 48, and 72 h. Cell proliferation assay were performed in triplicate.

### 2.6. Wound Healing Cell Migration Assay

For wound healing cell migration assay, USCs from donor 1 with passages 1–3 (3 × 10^4^) were seeded in 4-well culture plates with USC proliferation medium and incubated at 37 °C. At approximately 100% confluence, cells were treated with 0.4 mg/mL mitomycin C to stop cell growth. After 2 h of treatment, the cell layer was scratched with a 200 μL sterile pipette tip. The images of the migration were recorded using a light microscope (FV-1000 spectral, Olympus, Tokyo, Japan) immediately after scratching (0 h) and 24 h and 48 h later. The migration abilities of the cells were quantified by measuring the scratch widths at different time points using the ImageJ software (National Institutes of Health, Bethesda, MD, USA). Wound healing cell migration assay was carried out in triplicate.

### 2.7. Colony Forming Unit Fibroblast (CFU-F) Assay

For the colony forming unit fibroblast (CFU-F) assay, USCs from donor 1 within passage 5 were seeded at a density of 50–100 cells/cm^2^ in cell culture plates with USC proliferation medium and incubated at 37 °C for two weeks. The medium was changed once after one week. The incubated USCs were washed with 1× PBS, fixed with 4% formaldehyde, and stained with 0.5% crystal violet. Colonies containing over 50 cells were counted under a light microscope at ×40 magnification. CFU-F assay were performed in triplicate.

### 2.8. Differentiation of USCs

To test their differentiation capacity, USCs from donor 1 at passages 3–5 were induced into adipogenesis, chondrogenesis, and osteogenesis. Basically, the USCs differentiation were carried out based on the previously established protocol. For adipogenic differentiation [47], 4 × 10^4^ cells were grown in adipogenic differentiation media (DMEM low glucose consisted of 10% FBS, 1% penicillin/streptomycin, 1 μM dexamethasone, 500 μM 3-Isobutyl-1-methylxanthine (IBMX), 5 μg/mL insulin, and 1 × Insulin-Transferrin-Selenium (ITS, Thermo Fisher Scientific)) for 14 days; the entire medium was changed every 2 days. To assess differentiation to adipocytes, cells were fixed using 4% paraformaldehyde, washed with PBS, stained with oil red O at 25 °C for 4 h and then images were taken using a light microscope (FV-1000 spectral). For chondrogenic differentiation [48], 4 × 10^4^ cells were cultured in chondrogenic differentiation media (DMEM low glucose consisted of 2% FBS, 1% penicillin/streptomycin, 100 nM dexamethasone, 10 mM β-glycerophosphate, 50 μg/mL L-ascorbic acid, 10 pg/mL tumor growth factor (TGF)- β3, 1 mM sodium pyruvate, 40 μg/mL proline, and 1 × ITS) for 14 days; the entire medium was changed every 2 days. To assess chondrogenic differentiation, cells were fixed using 4% paraformaldehyde, washed with PBS, stained with Alcian blue at 25 °C overnight and then the images were taken using a light microscope (FV-1000 spectral). For osteogenic differentiation [49], 4 × 10^4^ cells were grown in osteogenic differentiation media (DMEM low glucose consisted of 10% FBS, 1% penicillin/streptomycin, 100 nM dexamethasone, 10 mM β-glycerophosphate, and 50 μg/mL L-ascorbic acid) for 14 days; the entire media was changed every 2 days. To assess osteogenic differentiation [50], cells were fixed using 4% paraformaldehyde, washed with PBS, stained with alizarin red S at 25 °C for 30 min, and then the images were captured using a light microscope (FV-1000 spectral). Each experiment was carried out in triplicate.

### 2.9. Generation of iPSCs from USCs and PBMCs

For the generation of USC-iPSCs, isolated USCs from donor from 1–5 within 5 passages were transduced with a CytoTune-iPS 2.0 Sendai Reprogramming Kit (Life Technologies, Carlsbad, CA, USA), which contains the four Yamanaka reprogramming factors, namely OCT4, SOX2, KLF4, and c-MYC, according to the manufacturer’s protocol. Briefly, 2 × 10^5^ cells were plated per well in Matrigel-coated six-well culture dishes with USC proliferation medium. At day 0, the cells were transduced overnight with Sendai viruses at the appropriate multiplicity of infection (MOI). Then, on the 4th day, the proliferation medium was replaced with the mTeSR1 medium (STEMCELL Technologies) and cells were cultured until colonies were formed. For the generation of PBMC-iPSC, cells within 3 passages were transduced with Sendai virus (SeV) at the appropriate MOI. Transduced cells were plated onto Matrigel-coated six-well plate culture with in mTeSR1 medium. Finally, the iPSC medium was replaced with mTeSR1 until colonies formed. We previously reported the detail of PBMC-iPSC clones [43].

For single cell sequencing for chromosome aneuploidy screening, USCs were cultured in 35 mm culture dishes using USC proliferation medium for 3 days. Cells were collected in 15 mL tubes, centrifuged at 1000 rpm for 5 min, and re-suspended in 1 mL PBS. Re-suspended single cells were harvested using picopipet (NEPAGENE, Ichikawa, Japan), and transferred into sterile 0.2 mL PCR tubes. Whole-genome amplification (WGA) and single cell next-generation sequencing (NGS)-based karyotypic analyses were performed by BMS Corporation (Gimpo, Republic of Korea).

### 2.10. Kidney Organoid Differentiation

For production of a hiPSC-derived kidney organoid, hiPSCs at passages 10–20 were dissociated with StemPro Accutase Cell Dissociation Reagent (Thermo Fisher Scientific), as described previously [51], and plated onto 24-well plates pre-coated with GelTrex Matrix (Thermo Fisher Scientific) in mTeSR1 with 10 μM Y-27632. The media was replaced with mTeSR1 + 1.5% GelTrex Matrix at 16 h for sandwiching hiPSCs. Then, the media was replaced at 60 h with 500 μL of 12 μM CHIR99021 (GSK3β inhibitor) in Advanced RPMI + Glutamax (Life Technologies), and at 96 h with RB (Advanced RPMI + Glutamax + B27 Supplement, from Life Technologies) and cultured for an additional two days. Afterwards, RB was changed every three days. Finally, we performed immuno-staining with lotus tetragonolobus lectin (LTL, Maravai LifeSciences, San Diego, CA, USA) and E-cadherin (Santa Cruz Biotechnology) to identify kidney organoid differentiation. Each experiment carried out in triplicate.

### 2.11. Immunocytofluorescence Staining

For the immunofluorescence staining, cells were fixed in PBS containing 4% paraformaldehyde for 20 min at RT and then washed three times with PBS. The cells were permeabilized with 0.1% Triton X-100 in PBS for 10 min at RT and then subjected to blocking with 1% bovine serum albumin (BSA) (MP Biomedicals, Santa Anacity, CA, USA) for 1 h at RT (25 °C). Afterwards, the USC-iPSCs were incubated with the primary antibody against stage-specific embryonic antigen (SSEA)-4 (1:100, Santa Cruz Biotechnology), OCT4 (1:100, Santa Cruz Biotechnology), NANOG (1:100, Santa Cruz Biotechnology), or SOX2 (1:100, Santa Cruz Biotechnology) overnight at 4 °C and then washed 3 times with PBS. The kidney organoids were incubated with the primary fluorescein isothiocyanate (FITC)-conjugated lotus tetragonolobus lectin (LTL) antibody (Maravai LifeSciences, San Diego, CA, USA) and E-cadherin antibody (Santa Cruz Biotechnology) to identify kidney organoid differentiation. Following this process, the cells were incubated with the corresponding secondary Abs (Alexa Fluor 488-conjugated donkey anti-rabbit IgG (H+L) (1:500, Invitrogen) or Alexa Fluor 546-conjugated rabbit anti-mouse IgG (H+L) (1:500, Invitrogen)) for 1 h at RT. Finally, cells were washed 3 times and stained with 4′,6-diamidino-2-phenylindole (DAPI, Thermo Fisher Scientific) for 10 min in order to visualize the nuclei. Fluorescent signals were examined using confocal microscope equipment (FV-1000 spectral).

### 2.12. HPC Differentiation

The iPSCs were differentiated into HPCs using the embryoid bodied (EB)-based HPC differentiation method [52,53]. For the differentiation, USC-derived iPSCs at passages 10–20 were treated with 3,2′-DHF or 3,4′-DHF and kept for 3 days on Matrigel-coated culture dishes in mTeSR1 medium. Afterwards, cells were transferred to ultra-low attachment surface dishes (Corning) with the same medium plus 10 μM Y-27632 for embryoid body (EB) formation over 6 days. Afterwards, human iPSCs differentiated into HPCs using the EBs. For HPCs differentiation, EBs were transferred to Matrigel coated dishes with the HPC differentiation medium (Iscove’s Modified Dulbecco’s Medium (Thermo Fisher Scientific) containing 20% FBS and 1% penicillin/streptomycin with 100 ng/mL SCF (Peprotech, Rocky Hill, NJ, USA), 10 ng/mL IL-3 (Peprotech), 10 ng/mL IL-6 (Peprotech), 20 ng/mL FLT3 (Peprotech), and 20 ng/mL BMP4 (Peprotech)) and half of the media was replaced with fresh media every 2 days throughout the differentiation period until day 21. HPC differentiation experiment carried out in triplicate.

### 2.13. HPC Colony-Forming Unit (CFU) Assay

CFU assays were performed using StemMACS HSC-CFU Media according to the manufacturer’s protocol as described previously (MACS miltenyi Biotec, Bergisch Gladbach, NRW, Germany) [54,55]. Briefly, 1 × 10^5^ HPCs differentiated from USC-iPSC-1 were mixed with 3 mL of StemMACS HSC-CFU Media (MACS miltenyi Biotec) by vortexing until a homogenous mixture was obtained and then the tubes were incubated at 25 °C for 10 min. The 1.1 mL aliquot of the mixture was then seeded using a sterile 5 mL syringe (KOVAX-SAFETY, Ansan, Republic of Korea) fitted to a 16-gauge blunt-end needle (STEMCELL Technologies) onto 35 mm petri dish. Finally, the 35 mm dishes were placed in a 100 mm dish. Besides, another 35 mm dish containing 3 mL sterile water without lid was placed in the 100 mm dish. All the dishes were incubated for 14–16 days in a humified incubator at 37 °C and 5% CO_2_ atmosphere. The medium is not changed. The colonies were visualized with morphology and color, and the number was measured by counting with a bright-field microscopy (FV-1000 spectral). Each experiment carried out in triplicate.

### 2.14. Statistical Analysis

Each experiment was repeated a minimum of three times. The data are presented as mean ± SEM. For statistical analysis, an unpaired two-tailed student’s’ t-test was performed between two groups using GraphPad Prism 7 software (GraphPad Software Inc., San Diego, CA, USA). *P* < 0.05 was considered to indicate a significant difference.

## 3. Results

### 3.1. Isolation and Characterization of USCs

**Figure 1:**
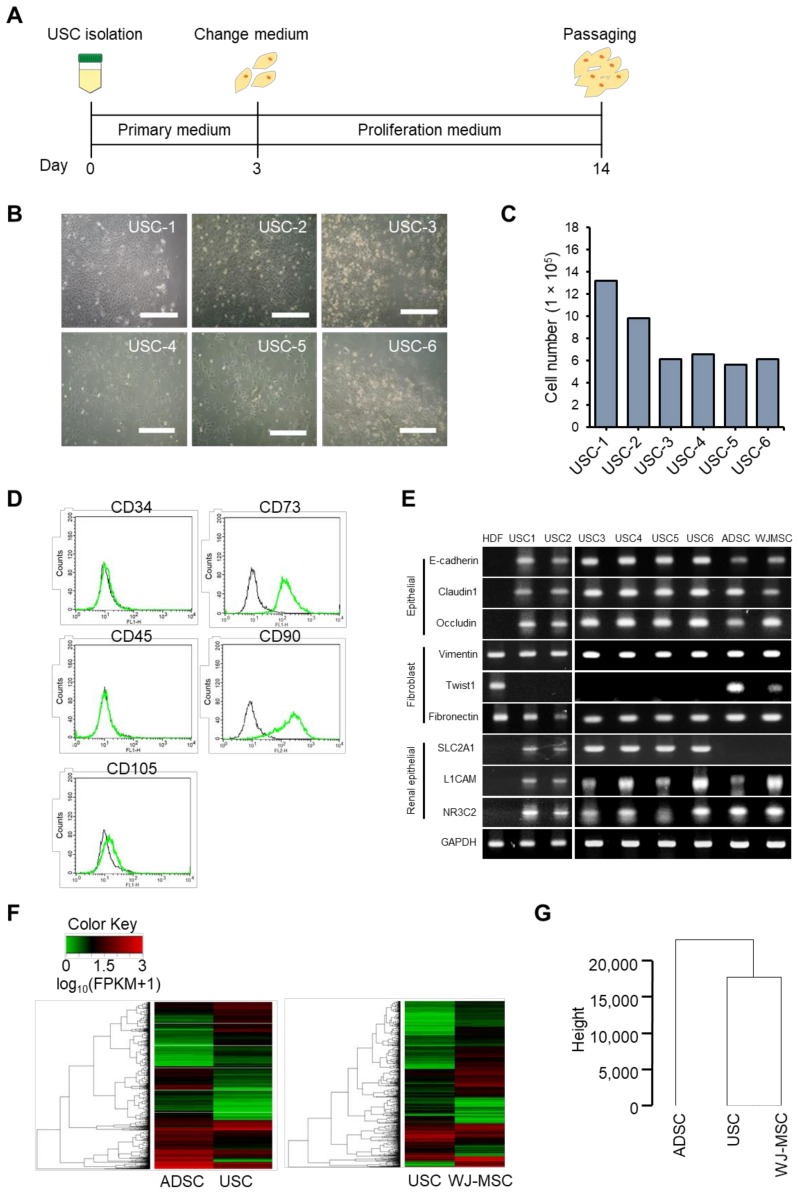
Characterization of urine stem cells (USCs). (**A**) Scheme of USC isolation. (**B**) Morphology of USCs from different donors after isolation (USC-1, 32-year-old male; USC-2, 50-year-old male; USC-3, 24-year-old male; USC-4, 22-year-old female; USC-5, 15-year-old female; USC-6, 20-year-old male). Scale bar: 400 µm. (**C**) Number of USCs at 14 days in the 6 urine samples. (**D**) Representative flow cytometric analysis of USC populations. (**E**) RT-PCR analysis of fibroblast markers (vimentin, twist1, fibronectin), epithelial markers (E-cadherin, claudin 1, occludin), renal epithelial markers (SLC2A1, L1CAM, NR3C2), and urothelial markers (CK13, CK20, UPK1a, UPK3a). (**F**) RNA sequencing of USCs, adipose derived stem cells (ADSCs), and Wharton’s jelly-derived mesenchymal stem cells (WJ-MSCs). Heatmap of hierarchical clustering of DEGs between of ADSCs, WJ-MSCs, and USCs (Fold change ≥2, *p*-value ≤ 0.05). (*G*) Dendrogram of hierarchical clustering group analysis based on gene expression profiles to show the similarities among the USCs, ADSCs, and WJ-MSCs.

We isolated USCs from human urine samples as previously described [44]. Cells were collected from 100–200 mL of urine from six different donors by centrifugation and initially cultured in primary cell culture media for 3 days, and then maintained in proliferation media for 11 days (Figure 1A). After 14 days of culture, colonies were formed for all samples (Figure 1B). The number of attached cells was counted by trypan blue exclusion. The total number of USCs in these samples was 5.6–13.2 × 10^5^ per urine sample (Figure 1C). USCs have multipotent MSC-like properties [56]. Thus, we assayed for the typical MSC surface markers in isolated USCs by flow cytometry. The positive MSC surface markers, CD73 and CD90, were highly expressed, while the negative markers, including CD34, CD45, and CD105, were not expressed (Figure 1D). RT-PCR amplification was used to examine the expression of epithelial, fibroblast, and renal epithelial markers (Figure 1E). Recently, renal epithelial markers have been reported to be highly expressed in USCs and renal proximal tubular epithelial cells [44]. We found that the expression of the epithelial markers E-cadherin, claudin 1, and occludin were higher in isolated USCs than in HDFs, as in ADSCs and WJ-MSCs. In addition, the fibroblast markers vimentin and fibronectin were expressed in HDFs, USCs, ADSCs, and WJ-MSCs, but USCs also expressed twist1 as reported previously [44]. The renal epithelial markers L1CAM and NR3C2 were not expressed in HDFs but were expressed in USCs, ADSCs, and WJ-MSCs. Specifically, SLC2A1 was shown to be express only in USCs. Overall, we successfully isolated USCs from six different donors, which was confirmed by the expression of MSC, fibroblast, and renal epithelial makers.

Although USCs are conveniently and non-invasively available for research and possible clinical use, their culture conditions, genetic characteristics, and differentiation mechanism have not been well established compared to other MSCs, including BMSCs, ADSCs, and WJ-MSCs. Here, we analyzed the genetic characteristics of USCs compared to ADSCs and WJ-MSCs, by RNA-seq (Figure 1F,G and Supplementary Figure S1). The heatmap of hierarchical clustering analysis visualized the different transcriptional profiles of ADSCs, USCs, and WJ-MSCs (Figure 1F). To determine the difference in gene expression profiling among the three MSC groups, we analyzed RNA Seq data of USCs, ADSCs, and WJ-MSCs by a dendrogram of hierarchical clustering analysis (Figure 1G). The results showed that mRNA expression profile of USCs is more similar to that of WJ-MSCs than that of ADSCs (Figure 1G). Next, we confirmed the DEGs among the three different MSC groups. Under the threshold of ≥2-fold change and *q*-value ≤ 0.05, we identified 2337 DEGs between ADSCs vs. USCs and 960 DEGs between WJ-MSCs vs. USCs (Supplementary Figure S1A–C).

Next, we categorized the 1004 upregulated DEGs in USCs compared to ADSCs into enriched categories according to GO analysis [57]. The three ontology categories (Biological Process, Cellular Component, and Molecular Function) were annotated and the top 10 categories that were significantly enriched (≥2-fold change, *q*-value ≤ 0.05) within each ontology were described. When we compared the number of genes belonging to each category, the genes in the categories of DNA metabolic process and DNA replication were most highly enriched in the “Biological Process” field (Supplementary Figure S1B, left panel). In terms of “Molecular Function”, genes associated with DNA replication origin binding, cadherin binding, and DNA secondary structure binding were most highly distributed (Supplementary Figure S1B, middle panel). In the “Cellular Component” section, genes involved in the chromosome/centromeric region, spindle, and microtubule organizing center were counted as the top three categories (Supplementary Figure S1B, right panel). A total of 254 upregulated DEGs in USCs compared to WJ-MSCs were also enriched into categories according to GO analysis. The serine family amino acid biosynthetic process, pri-miRNA transcription from RNA polymerase II promoter, and cell-substrate junction assembly were the predominant categories in “Biological Process” (Supplementary Figure S1C, left panel). Transcripts coding for proteins associated with calcium: sodium antiporter activity, neutral amino acid transmembrane transporter activity, and cyclic-nucleotide phosphodiesterase activity were enriched as the top three categories in the “Molecular Function” ontology (Supplementary Figure S1C, middle panel). In the case of “Cellular Component”, genes belonging to intermediate filament, axolemma, and early endosome membrane were highly enriched (Supplementary Figure S1C, right panel). WJ-MSCs have higher proliferation, self-renewal, and differentiation and lower senescence abilities than ADSCs and BMSCs [58,59]. Interestingly, USCs showed a closer gene profile in the dendrogram of hierarchical clustering with WJ-MSCs than ADSCs. In fact, USCs showed higher self-renewal and expansion capability, multi-lineage differentiation capability, telomerase activity, and telomere length compared to ADSCs and BMSCs [23]. Thus, we expect to be able to use these gene analysis results to analyze the mechanism and cell differentiation function, which warrants further studies. This genetic analysis with RNA seq data suggested that USCs are closer to WJ-MSCs than ADSCs.

### 3.2. Y-27632 and Matrigel Enhance USC Isolation Efficiency

**Figure 2:**
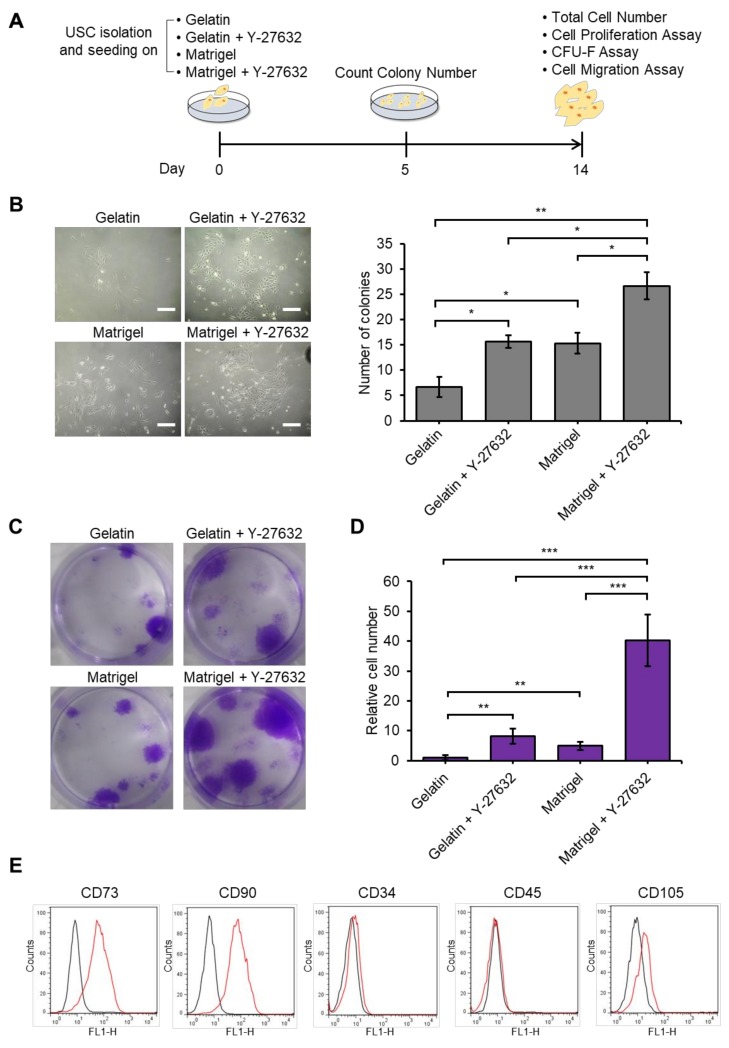
Y-27632 and Matrigel markedly increased the isolation efficiency of USCs. (**A**) Schematic of the USC isolation method using gelatin, Y-27632, Matrigel. (**B**) Representative phase-contrast of USCs isolated using gelatin, gelatin + Y-27632, Matrigel, and Matrigel + Y-27632 (left panel). Number of USC (6 donor) colonies obtained using gelatin, gelatin + Y-27632, Matrigel, and Matrigel + Y-27632 (right panel) in the well of a 12 well culture plate. Scale bar: 200 µm. (**C**) Images of USCs treated with gelatin, gelatin + Y-27632, Matrigel, and Matrigel + Y-27632 and stained with crystal violet after 14 days. (**D**) Relative cell number of isolated from USCs (6 donor) after 14 days deduced with the number of isolated cells in gelatin-only plate set to 1. Asterisks indicate significant differences. (**E**) Flow cytometric analysis of USC surface marker expression on the USCs of Y-27632 + Matrigel group (black line: isotype control (Con), red line: USC specific marker). *n* = 3 biological samples. (* *p* < 0.05, ** *p* < 0.01, *** *p* < 0.001).

Improved USC isolation methods are necessary to obtain sufficient numbers of USCs for clinical application and research use. Previous USC isolation methods employed gelatin, one of the ECM proteins, as a coating material on a cell culture dish [44,56,60]. In this study, we tested Matrigel compared to gelatin as a coating agent. In addition, we studied the effect of Y-27632, a ROCK inhibitor, on the yield of the isolation because Y-27632 also increases the yield of post-thaw viability of cryopreserved and subcultured adult stem cells and PSCs [30,32,61]. Thus, we examine the effect of Y-27632 with a gelatin or Matrigel coating on colony formation and the total number of cells, respectively, after 14 days of culture (Figure 2A). The number of USC colonies after 5 days in culture was 2–3-fold higher in the gelatin-coated dish supplemented with Y-27632 than that in the only gelatin-coated group. Similarly, 2~3-fold more colonies were developed in the only Matrigel-coated dish group compared to the only gelatin-coated group after 5 days of culture. Interestingly, colony formation was 5-fold higher in the Matrigel-coated dish supplemented with Y-27632 compared to the gelatin-coated group (Figure 2B). After 14 days of culture, USCs were stained using crystal violet for confirming the colony formation efficiency (Figure 2C), and the cells were counted (Figure 2D). The cell numbers were significantly increased by approximately 10-fold with Y-27632 treatment in the gelatin-coated dish. In addition, the cell number in the Matrigel-coated dish was significantly increased compared to the gelatin-coated dish. The number of cells increased by approximately 40-fold upon Y-27632-treatment in Matrigel-coated dishes compared to the gelatin-coated dishes without Y-27632 treatment (Figure 2D). Therefore, treatment of Y-27632, the use of Matrigel, or their combination significantly increased the yield of harvested USCs after isolation compared to gelatin coating without Y-27632 treatment. USCs in the Y-27632 + Matrigel group had strong CD73 and CD90 surface expression and very weak CD105 but not CD34 and CD45 expression. These marker expression patterns suggested that USCs in the Y-27632 + Matrigel group maintain USC character like the cells in gelatin only group (Figure 2E).

### 3.3. Y-27632 and Matrigel Enhance USCs Properties

**Figure 3:**
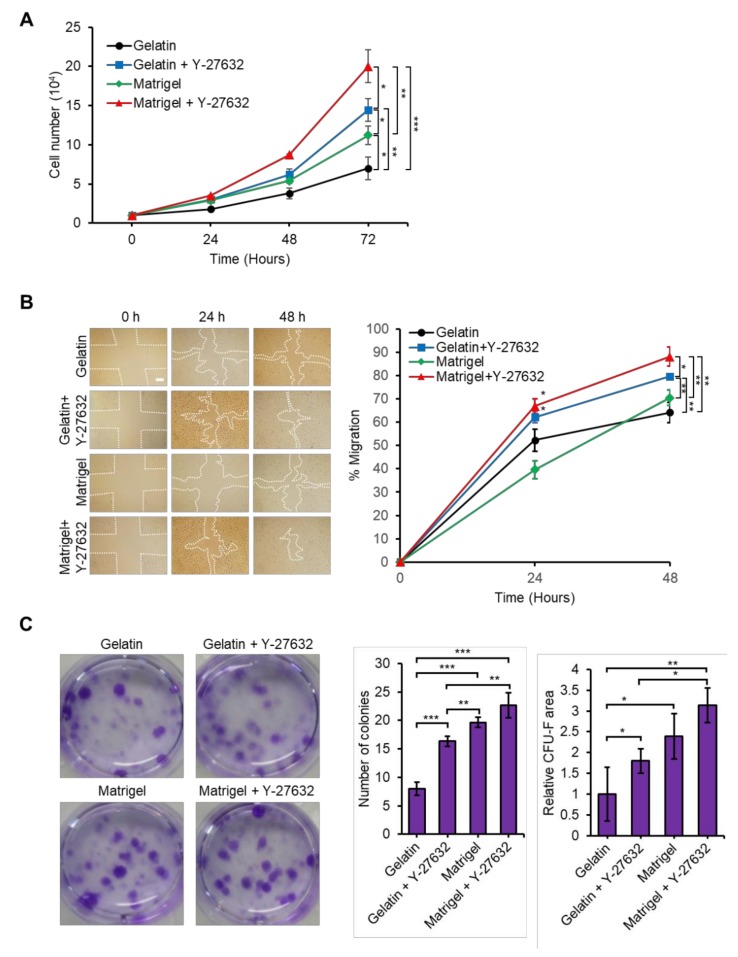
Y-27632 and Matrigel enhances the properties of USCs. (**A**) Growth curve of gelatin, gelatin + Y-27632, Matrigel, Matrigel + Y-27632 treated USCs at different time points. (**B**) Wound healing assay. Cell migration was assessed by the recovery of the scratch. Gelatin, gelatin + Y-27632, Matrigel, Matrigel + Y-27632 treated USCs, respectively. Scale bar: 200 µm. (**C**) Colony forming unit fibroblast (CFU-F) assays. At each group, representative plates of CFU-F colonies stained with crystal violet are shown (left panel). CFU-F colonies were counted after culture for 14 days. Asterisks indicate significant differences. The data are presented as fold changes compared with the gelatin group (right panel). *n* = 3 biological samples. (* *p* < 0.05, ** *p* < 0.01, *** *p* < 0.001).

Next, we compared the proliferation, migration, and colony forming ability of USCs at 14 days in culture with or without Y-27632 treatment in gelatin- or Matrigel-coated plates as described in Figure 3. We isolated USCs from gelatin, gelatin + Y-27632, Matrigel, and Matrigel + Y-27632 plates and seeded them on non-coated cell culture dishes to compare the proliferation rates of USCs. After 72 h of culture, the cell numbers of USCs isolated from gelatin + Y-27632, Matrigel-coated, and Matrigel + Y-27632 plates were significantly higher than those of USCs isolated from gelatin-coated plates. In particular, the growth rate of the Matrigel + Y-27632 group was increased to more than 3-fold as compared to the gelatin (control) group at 72 h (Figure 3A).

The migration of MSCs is closely related with their therapeutic efficacy [62,63,64]. To examine the migration ability of USCs, we performed a wound healing assay. USCs, isolated from gelatin + Y-27632 and Matrigel + Y-27632 groups, showed a significantly increased cell migration rate compared to USCs from the gelatin group at 48 h. However, USCs from the Matrigel coating group did not show a significant difference in migration compared to USCs from the gelatin group (Figure 3B).

The colony formation ability of mesenchymal stem cells is an important characteristic reflecting the quality of self-renewal [65]. Thus, we performed CFU-F assays for assessing the colony forming ability of USCs isolated from gelatin, gelatin + Y-27632, Matrigel, and Matrigel + Y-27632 plates (Figure 3C). The number and size of colonies of USCs isolated from gelatin + Y-27632, Matrigel, and Matrigel + Y-27632 were higher than those of USCs obtained from gelatin-coated plates (Figure 3C). Therefore, USCs isolated using Matrigel coating and/or Y-27632 treatment significantly improved cell proliferation, migration, and self-renewal ability compared to USCs isolated using only a gelatin coating.

### 3.4. Y-27632 and Matrigel Enhance Differentiation of USCs

**Figure 4:**
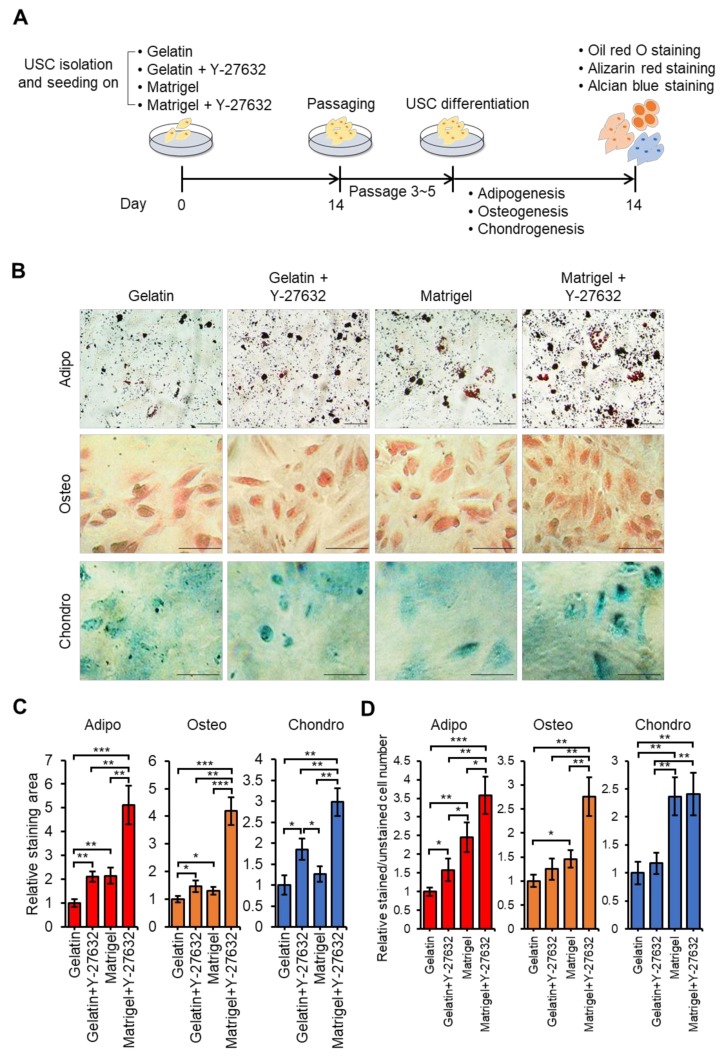

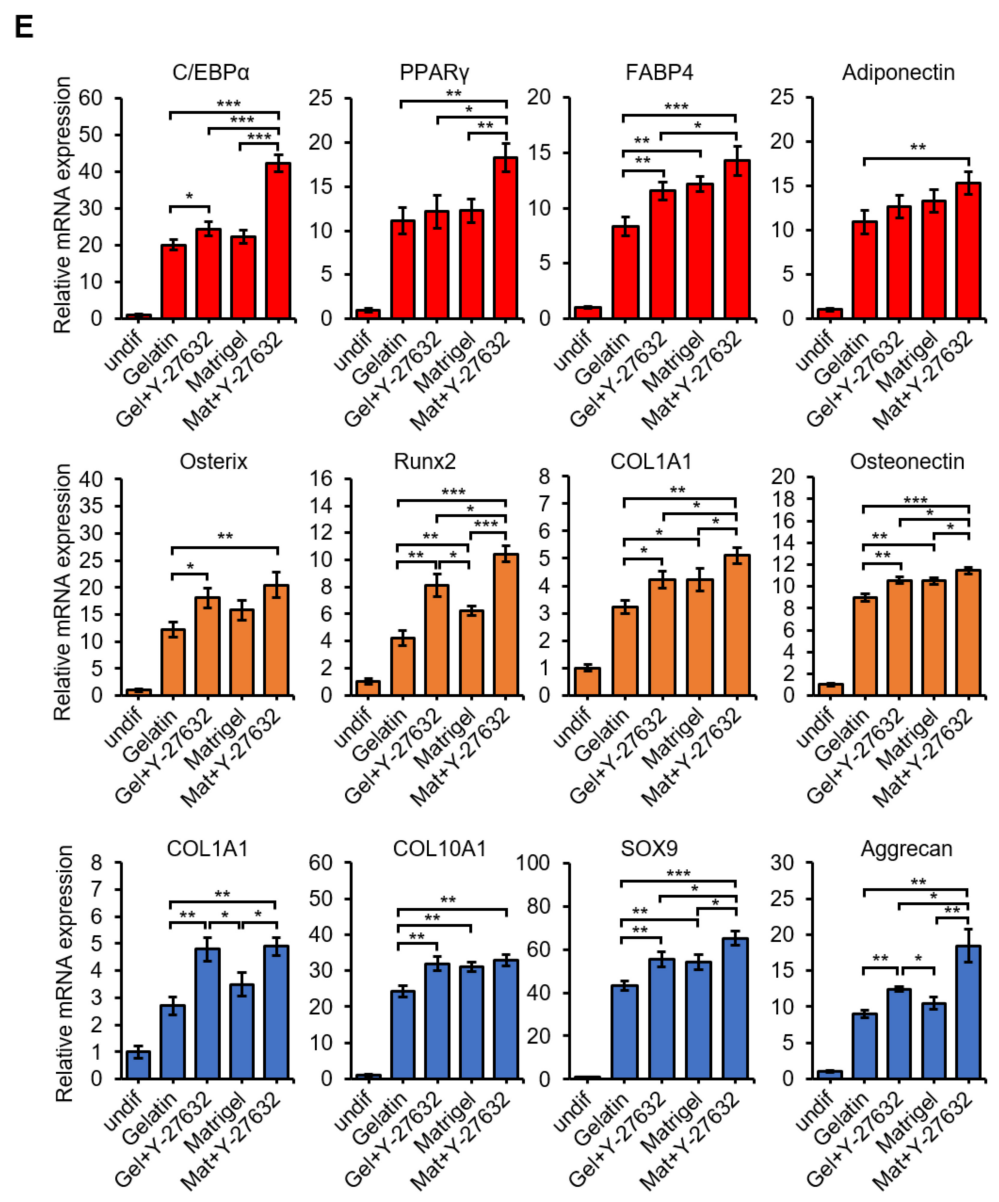
The effect of Y-27632 and Matrigel in the adipogenic, osteogenic, and chondrogenic differentiation of USCs. (**A**) Schematic of the USC differentiation procedure with gelatin and Matrigel. (**B**) Differentiation properties of USCs. Oil red O staining for adipogenesis, Alizarin red S staining for osteogenesis, and Alcian blue staining for chondrogenesis. Scale bar: 50 μm. (**C**) Relative staining area of Oil red O, Alizarin red S and Alcian blue with gelatin only data set to 1. (**D**) Relative ratio of stained to unstained cell number of Oil red O, Alizarin red S and Alcian blue. The ratio of gelatin control group was set to 1 for all graphs. (**E**) mRNA expression profiles of USCs differentiated to adipogenic, osteogenic, and chondrogenic cells. Adipogenic differentiation markers: C/EBPα, PPARγ, FABP4, and adiponectin. Osteogenic differentiation markers: Osterix, Runx2, COL1A1, and osteonectin. Chondrogenic differentiation markers: COL1A1, COL10A1, SOX9, and aggrecan. The data are presented as fold changes compared with the undif. *n* = 3 biological samples. (* *p* < 0.05, ** *p* < 0.01, *** *p* < 0.001).

USCs have great potential to differentiate into different types of cells [60]. To evaluate the differentiation potential of our isolated USCs, which were cultured on gelatin, gelatin + Y-27632, Matrigel, and Matrigel + Y-27632, we assayed their adipogenic, chondrogenic, and osteogenic differentiation capacities (Figure 4A). After 14 days of differentiation in culture, adipocyte differentiation was identified by oil red O staining assay, osteoblast differentiation was verified by Alizarin red S staining assay, and chondrogenic differentiation was confirmed by Alcian blue staining (Figure 4B).

Relative oil red O staining was significantly more intense and had higher stained per unstained cell ratio in the gelatin + Y-27632, Matrigel only, and Matrigel + Y-27632 groups than in the gelatin only group (Figure 4C,D). USCs from Matrigel + Y-27632 plates showed the greatest adipogenic potential among the experimental groups, which was at least 5-fold higher than that of USCs from the gelatin-only group in staining area and 3.5-fold in a ratio of staining to unstained cells. Alizarin red S staining was 4-fold denser in the Matrigel + Y-27632 group than in the gelatin group and more than 2.5-fold in a ratio of stained to unstained cells, which was the highest among the other USC groups. In the chondrogenesis assay, the gelatin + Y-27632 and Matrigel + Y-27632 groups showed significantly higher Alcian blue staining area than the gelatin group, whereas the Matrigel group did not show significant increase. However, the ratio of stained to unstained cells was significantly increased in Matrigel + Y-27632 and Matrigel group but not in the gelatin + Y-27632. The Matrigel + Y-27632 group showed the highest Alcian staining level and stained cell ratio, among experimental groups which meant the Matrigel + Y-27632 group has the highest chondrogenic differentiation capacity.

To confirm the differentiation at the gene expression level, we analyzed adipogenic, osteogenic and chondrogenic differentiation markers using real time-PCR (Figure 4E). The expression of the adipocyte markers C/EBPα, PPARγ, FABP4, and adiponectin was examined in each USC group. Cells differentiated from USCs grown in Matrigel + Y-27632 had the highest increase in adipocyte marker expression levels. The expression of osteogenic differentiation markers, such as Osterix, Runx2, COL1A1, and osteonectin, was higher in USCs from the Matrigel + Y-27632 group than in USCs from all other groups. Similarly, the expression of chondrogenic differentiation markers, such as COL1A1, COL10A1, SOX9, and aggrecan, was significantly higher in the USCs from the Matrigel + Y-27632 groups than USCs from all other groups. Collectively, we confirmed the better differentiation potential of USCs isolated using Y-27632, Matrigel, and Matrigel + Y-27632 plates than USCs grown in gelatin-only plates. USCs isolated with Matrigel + Y-27632 showed the greatest differentiation properties.

### 3.5. Flavonoids Increase the Isolation Efficiency of USCs

**Figure 5:**
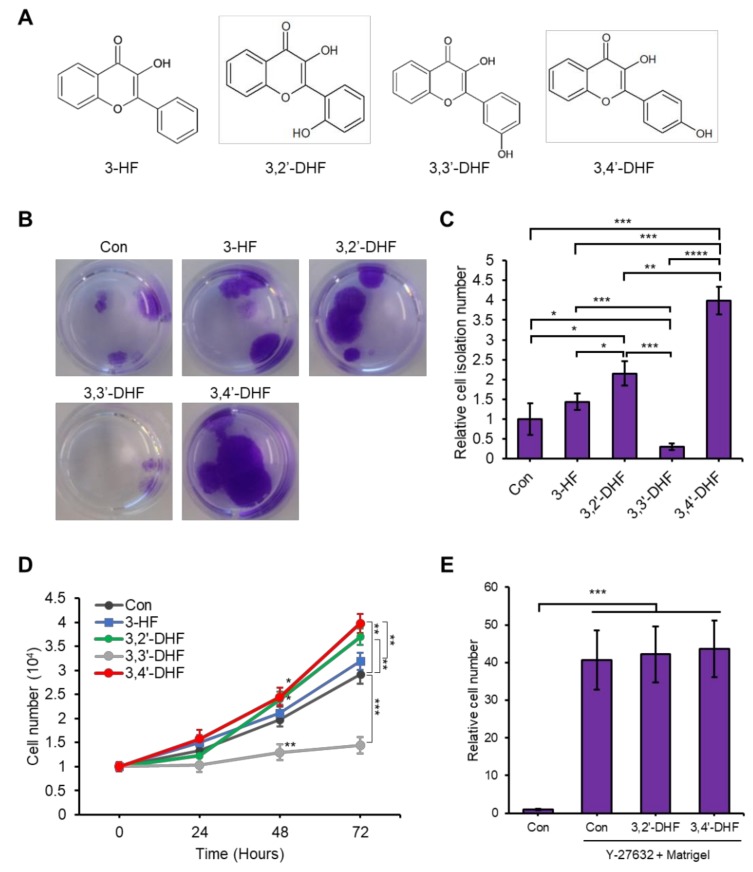
Effects of 3-hydroxyflavone (3-HF), 3,2′-dihydroxyflavone (3,2′-DHF), 3,3′-DHF, and 3,4′-DHF on USC isolation efficiency. (**A**) Structures of the flavonoids 3-HF, 3,2′-DHF, 3,3′-DHF, and 3,4′-DHF. (**B**) Pictures of USCs treated with 3-HF, 3,2′-DHF, 3,3′-DHF, and 3,4′-DHF and stained with crystal violet after 14 days. (**C**) Relative cell number of isolated USCs after 14 days to the cell number of control (no treatment on gelatin coated plate) set to 1. (**D**) Growth curve of 3-HF-, 3,2′-DHF-, 3,3′-DHF-, and 3,4′-DHF-treated USCs at different time points. (**E**) Relative cell number of isolated USCs after 14 days incubation treated with 3,2′-DHF, 3,4′-DHF and/or Y-27632 + Matrigel compared to the number of cells of gelatin group control set 1. *n* = 3 biological samples. (* *p* < 0.05, ** *p* < 0.01, *** *p* < 0.001, **** *p* < 0.0001).

The flavonoids 3-hydroxyflavone (3-HF), 3,2′-dihydroxyflavone (3,2′-DHF), 3,3′-DHF, and 3,4′-DHF enhanced the proliferation and differentiation of stem cells (Figure 5A) [35,41,66]. Here, we also applied the flavonoids to examine their effect on the isolation efficiency and proliferation capacity of USCs on basic USC isolation protocol (gelatin group). We added 5 μM 3-HF, 3,2′-DHF, 3,3′-DHF, or 3,4′-DHF in culture media during the isolation of USCs in gelatin coated plate. Among the tested flavonoids, 3,2′-DHF and 3,4′-DHF significantly increased isolation efficiency compared to the non-treated gelatin-coated control (Figure 5B,C). However, treatment with 3,3′-DHF decreased the number of harvested cells. We then measured the proliferation rate for 72 h of the USCs isolated with or without flavonoids. Cells isolated with 3,4′-DHF or 3,2′-DHF showed significantly enhanced proliferation rate compared to the control gelatin group (Figure 5D). However, a fold increase in the isolation efficiency and proliferation capacity of the isolated USCs with flavonoid treatment is much lower than with Y-27632 + Matrigel treatment (Figure 3). We then examined the synergistic effect of flavonoids and Y-27632 + Matrigel treatment in USC isolation (Figure 5E). However, 3,2′-DHF or 3,4′-DHF treatment did not increase the isolated cell number on the Y-27632 + Matrigel group. In summary, treatment with 3,2′-DHF and 3,4′-DHF increased the number of harvested cells and improved the proliferation capacity of the isolated USCs on gelatin-coated control but did not show synergy with Y-27632 + Matrigel treatment.

### 3.6. Generation of USC-iPSCs and Their Characterization

**Figure 6:**
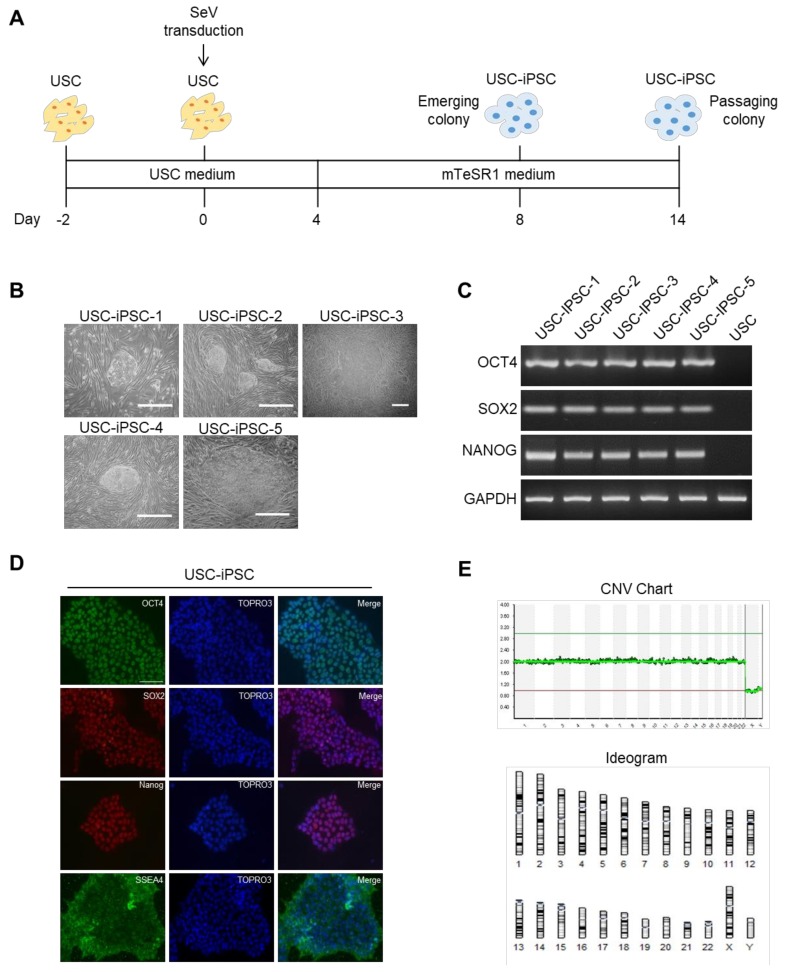
Generation of USC-derived induced pluripotent stem cells (iPSCs). (**A**) Schematic representation of hiPSC generation from USCs using SeV. (**B**) Phase contrast of USC-iPSCs (USC-iPSC-1, 32-year-old male; USC-iPSC-2, 50-year-old male; USC-iPSC-3, 24-year-old male; USC-iPSC-4, 22-year-old female; USC-iPSC-5, 20-year-old male). Scale bar: 200 µm. (**C**) RT-PCR analysis showing expression of *SOX2*, *NANOG*, and *OCT4* in USC-iPSCs. (**D**) Representative immunofluorescence analysis of USC-iPSCs showing the expression of human pluripotent stem cell-specific markers, such as NANOG, SOX2, OCT4, and SSEA4. Scale bar: 40 μm. (**E**) Single-cell array-based comparative genomic hybridization (aCGH) sequencing for USC-iPSC-1 chromosome abnormalities. (**F**) RNA sequencing of peripheral blood mononuclear cells (PBMCs), USCs, embryonic stem cells (ESCs), USC-iPSCs, and PBMC-iPSCs. Heatmap of hierarchical clustering of differentially expressed genes (DEGs) between of PBMC-iPSCs and USC-iPSCs (Fold change ≥ 2, *p*-value ≤ 0.05). (**G**) Dendrogram of hierarchical clustering group analysis based on gene expression profiles to show the similarities among the PBMCs, USCs, ESCs, USC-iPSCs, and PBMC-iPSCs.

We evaluated the capability of our USCs to be reprogramed into iPSCs. SeV has been used as vector for gene therapy since it has no risk of being integrated into the host genome and is nonpathogenic to humans [67]. We performed SeV transduction of USCs from 5 different donors and cultured them in USC medium for 4 days; subsequently, USCs were cultured for 12 days in mTeSR1 medium (Figure 6A). After 12 days of culture, embryonic stem (ES)-like colonies were observed. (Figure 6B). Then, in order to ensure that the iPSCs were successfully generated, the expression of iPSC markers was confirmed by RT-PCR. In comparison with USCs, the expression of OCT4, SOX2, and NANOG was higher in USC-iPSCs (Figure 6C). The expression of the protein markers OCT4, SOX2, NANOG, and SSEA-4 in USC-iPSC was also observed by immunostaining (Figure 6D). NGS-based karyotype analysis of single cells was performed according to a single cell NGS-based 24-chromosome aneuploidy screening protocol [31,32]. No chromosomal aberrations were identified in 3 USC-iPSC clones, which were used in subsequent experiments (Figure 6E and Supplementary Figure S3).

To analyze the mRNA expression of PBMCs, USCs, ESCs, USC-iPSCs, and PBMC-iPSCs, RNA sequencing was performed. The mRNA expression of PBMC-iPSCs and USC-iPSCs, comparatively visualized by hierarchical heat map, showed that a substantial number of genes were differentially expressed between the two cell types (Figure 6F,G). Hierarchical clustering analysis of RNA-seq data from PBMCs, USCs, ESCs, USC-iPSCs, and PBMC-iPSCs showed that the gene expression pattern of USC-iPSCs was most closely clustered with PBMC-iPSCs and next with ESCs (Figure 6G). The similarity in the mRNA expression pattern of USC-iPSCs and other pluripotent stem cells (PBMC-iPSCs and ESCs) suggested successful reprogramming into PSCs. The gene expression pattern of USC-iPSCs was more closely clustered with USCs than PBMCs, which may reflect the maintenance of epigenetic memory after the reprogramming processes (Figure 6G). Compared to the PBMC-iPSCs, there were 272 more upregulated DEGs and 489 more downregulated DEGs in USC-iPSCs (Supplementary Figure S2A). In particular, among the upregulated DEGs, adult kidney-related tissue protein expression genes were the most abundant (Supplementary Figure S2B). Overall, the mRNA expression profile of USC-iPSCs suggested similar gene expression to that of other pluripotent stem cells and high expression of some the adult kidney genes.

### 3.7. Differentiation Potential of USC-iPSCs into Kidney Organoids and HPCs.

**Figure 7:**
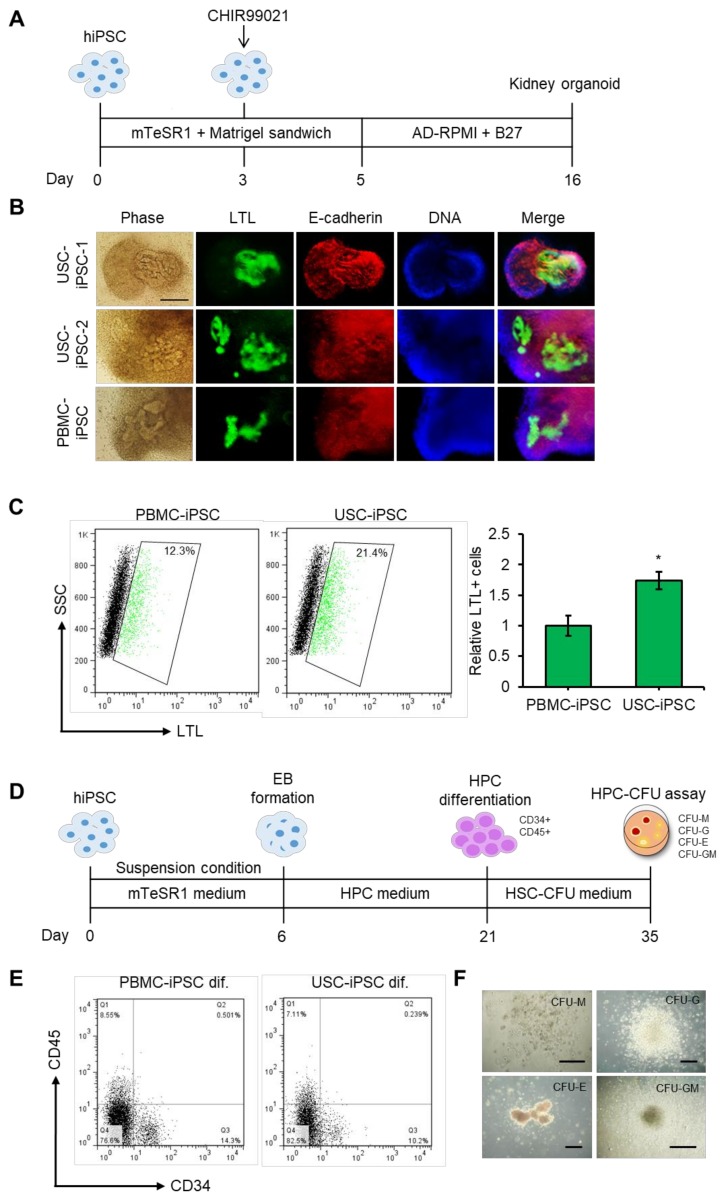
Differentiation of USC-iPSCs. (**A**) Schematic representation of USC-hiPSC-derived kidney organoids. (**B**) Representative images showing changes in cell morphology and lotus tetragonolobus lectin (LTL) proximal tubule marker expression during 16 days of kidney organoid differentiation. Scale bar: 100 µm. (**C**) Flow cytometry analysis of LTL binding in PBMC-iPSC and USC-iPSC derived kidney organoids as a percentage of the total population. The data are presented as fold changes compared with the PBMC-iPSC. (**D**) Schematic representation of USC-iPSC derived HPCs. (**E**) Flow cytometry analysis of HPC markers of differentiated USC-iPSC derived HPCs. (**F**) Colony forming unit (CFU) analysis of USC-hiPSC derived HPCs. M, Megakaryocyte; G, Granulocyte; E, Erythroid. *n* = 3 biological samples. (* *p* < 0.05).

USCs are likely to be kidney-derived cells [23]. Differentiation of iPSCs into their original specific type of cells is much easier because of epigenetic memory of their original cells in iPSCs [68]. Therefore, we generated kidney organoids from USC-iPSCs to test their differentiation potential. To generate kidney organoids, USC-iPSCs and PBMC-iPSCs were subjected to differentiation as described previously (Figure 7A) [51]. The generation of kidney organoids was confirmed with the expression of proximal tubule marker LTL and a distal tubule marker, E-cadherin, by immunostaining (Figure 7B). Interestingly, the proportion of LTL positive cells was higher in USC-iPSC-derived kidney organoids than in PBMC-iPSC-derived organoids (Figure 7C). We also successfully differentiated USC-iPSCs into HPCs. First, USC-iPSCs were differentiated into EB for six days and further differentiated to HPCs for 15 days (Figure 7D). At day 21, the expression levels of HPC markers CD34+ and CD45+ were examined using flow cytometry (Figure 7E). In our results, approximately 10.2% CD34+, 7.1% CD45+, and 0.2% CD34+ CD45+ cell populations were detected in USC-iPSC-1 group (Figure 7E) and around 12.2% CD34+, 4.2% CD45+, and 2.9% CD34+/CD45+ cell populations in the USC-iPSC-2 group (Supplementary Figure S4). Although the differential differentiation efficiency was obtained with the different USC lines, we could observe the apparent differentiation into CD34+ and CD45+ HPCs with the different USC-iPSC lines. Of note, the proportion of CD34+ and CD45+ cells were lower in cells differentiated from USC-iPSCs than in cells differentiated from PBMC-iPSCs. Subsequently, cells were further differentiated with HSC-CFU medium for 14 days. Various types of hematopoietic lineage colonies were identified after the HPC-CFU assay, which suggested the hematopoietic stemness of differentiated cells (Figure 7F). Overall, USC-iPSCs have the potential to differentiate into kidney organoids and HPCs. Our results imply that isolated USCs have great potential to generate iPSCs, which can be differentiated into HPCs, which might be applied in regenerative medicine.

### 3.8. Flavonoid Treatment of USC-Derived iPSCs Enhances Capacities to Differentiate into HPCs.

**Figure 8:**
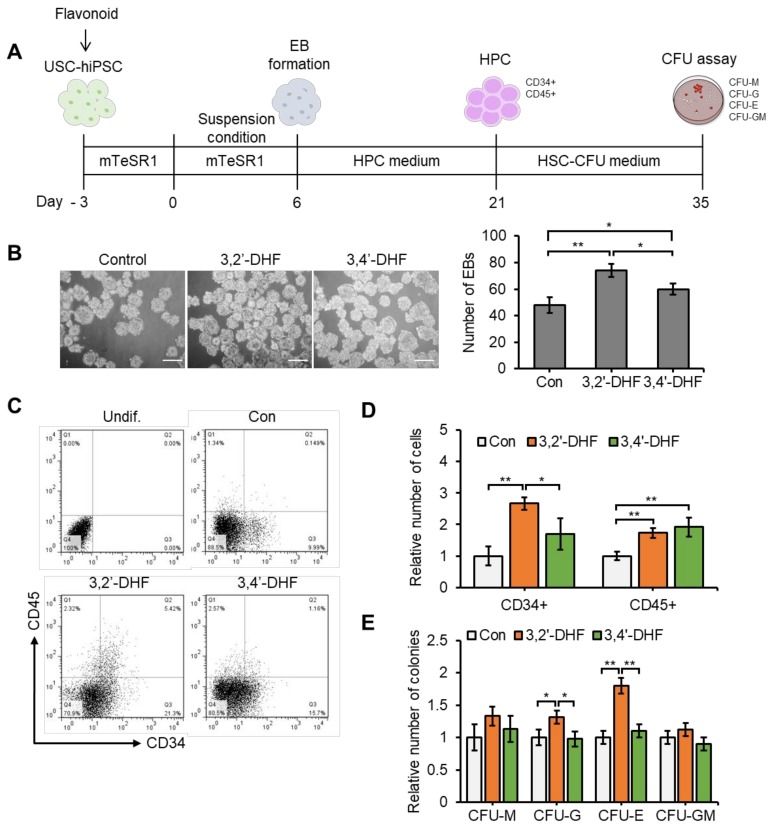
Effects of 3,2′-DHF and 3,4′-DHF on USC-iPSCs differentiation to HPCs. (**A**) Schematic of the HPC differentiation process. (**B**) Effects of 3,2′-DHF and 3,4′-DHF on USC-iPSC EB formation. (**C**) Representative flow cytometry dot plots of the hematopoietic markers observed during hiPSC differentiation of the control and 3,2′-DHF- and 3,4′-DHF-treated cells. (**D**) Relative number of CD34+ and CD45+ cells during hematopoietic differentiation. The data are presented as fold changes compared with the Con group. (**E**) Colony forming unit (CFU) potential of control, 3,2′-DHF- and 3,4′-DHF-treated USC-iPSCs. The data are presented as fold changes compared with the Con group. *n* = 3 biological samples. (* *p* < 0.05, ** *p* < 0.01, *** *p* < 0.001).

Next, we investigated the effects of flavonoids on the differentiation of iPSCs into HPCs (Figure 8). We applied 3,2′-DHF and 3,4′-DHF during differentiation of USC-iPSCs to HPCs for 3 days before initiation of EB formation. Then, cells were allowed to differentiate into EBs for 6 days and then into HPCs for 14 days (Figure 8A). Interestingly, the number of EBs was significantly increased in the 3,2′-DHF- and 3,4′-DHF-treated groups compared to the untreated group (Figure 8B). After a total of 21 days of differentiation, we measured the frequency of HPC surface markers among the untreated control cells, and the 3,2′-DHF- and 3,4′-DHF-treated cells (Figure 8C). There was significant increase in the number of CD34+ and CD45+ cells in the groups treated with 3,2′-DHF and 3,4′-DHF compared to the control group (Figure 8D). After further differentiation of 5 × 10^5^ cells in the HPC-CFU medium, the 3,4′-DHF-treated cells showed no significant difference in CFU formation compared to the control, but 3,2′-DHF-treated cells produced significantly increased numbers of colony forming unit-granulocyte (CFU-G) and colony forming unit-erythroid (CFU-E) (Figure 8E). These results suggest that 3,2′-DHF and 3,4′-DHF promote the differentiation of USC-iPSCs into hematopoietic lineage cells.

## 4. Discussion

MSCs are located in various tissues throughout the human body [69]. MSCs can differentiate into various cell lines, including osteocytes and chondrocytes, in response to environmental signals [70]. Multipotent MSCs are currently considered as the most promising cell source for cell therapeutic applications. USCs, one kind of MSC, offer apparent advantages over ADSCs and BMSCs given their simple and non-invasive isolation method and multi-differentiation potential [23,60]. Therefore, USCs could be the best source of cells for personalized cell therapy if appropriate scalable cell preparation methods are established. We successfully isolated USCs from 6-donors (Figure 1). The absence of Twist1 expression in USCs was interesting because Twist1 controls MSC allocation during development [71,72]. Silencing Twist1 enhances osteogenic differentiation of ADSCs [73]. Specific expression of the renal epithelial maker SLC2A1 in USCs from all donors suggests that the origin of USCs may be the renal proximal tubular epithelium [44]. Such distinct expression of Twist1 and SLC2A1 in USCs compared to other mesenchymal cells determines their specific properties. Gene expression profiles of USCs compared with WJ-MSCs and ADSCs also showed substantial differences in gene expression (Figure 1). Ontology analysis of DEGs revealed that USCs have enriched gene expression related with cell cycle and DNA replication compared to the ADSCs (Supplementary Figure S1). This result corresponds to the already reported higher proliferative properties of USCs compared to ADSCs [22]. Our results indicated that USCs showed gene expression profiles closer to WJ-USCs than ADSCs.

In recent regenerative medicine, stem cells have shown great potential as a treatment for various diseases. Expandability and differentiation capabilities are key properties of stem cells in the field of cell therapy and tissue engineering. In our study, we demonstrated that treatment with Y-27632, Matrigel, and flavonoids improved the efficiency of USC isolation and proliferation and differentiation capacities of isolated USCs. The ROCK inhibitor Y-27632 and Matrigel have been proposed to enhance survival or proliferation in hPSC culture [61,74,75]. Y-27632 has also been reported to improve cell recovery of cryopreserved hPSCs [76]. Furthermore, treatment of hPSCs with Y-27632 did not affect the cell karyotype and gene expression required for self-renewal nor did it affect the differentiation of hPSCs into other cells [77]. ROCK inhibitors have also been shown to promote proliferation, viability, and differentiation of MSCs [31,78,79,80]. Matrigel has recently been used to replace the use of mouse embryonic fibroblasts (MEFs) in hPSC culture [81]. USCs have been isolated in the presence of only a gelatin-coated layer [24,44,56], which enhances their attachment during the initial step of their preparation. In our study, we examined the effect of Y-27632, Matrigel, and their combination on USC isolation for the first time. We demonstrated that the use of Matrigel and Y-27632 in USC isolation dramatically improved the number of attached colonies (more than 4-fold) and the total isolation efficiency (approximately 40-fold) compared to the use of gelatin only. In addition, isolated USCs treated with Y-27632 and Matrigel showed improved proliferation, self-renewal ability, and migration ability, which are the critical properties for tissue regeneration [80]. MSCs can differentiate into mesodermal cell lineages, including osteocytes and chondrocytes. We examined chondrogenic and osteogenic differentiation using isolated USCs. Particularly, USCs isolated from Matrigel and Y-27632 showed higher differentiation ability. Overall, Y-27632 and Matrigel provided the highest yield of isolated USCs.

We successfully reprogrammed USCs to USC-iPSCs using SeV reprogramming vectors. Previous studies have shown that USCs originate from the kidney [60]. iPSCs have epigenetic memories of their source tissue that affect their differentiation efficiency and fidelity to other cells [82,83]. Our RNA-seq results also clearly showed that the gene expression profile of USCs is more similar to USC-iPSCs than PBMC-iPSCs. DEGs highly expressed in USCs-iPSC were enriched in kidney genes (Supplementary Figure S2). Moreover, our results showed that a greater number of proximal tubule positive cells were found in kidney organoids differentiated from USC-iPSCs than from PBMC-iPSCs (Figure 7B,C). However, differentiation of USC-iPSCs to HPCs was less effective compared to differentiation of PBMC-iPSCs to HPCs (Figure 7E and Supplementary Figure S4). Collectively, these results showed that USCs have genetic memory of the original cells, which could drive a stronger tendency to differentiate into cell lineages adjacent to the original cells. Successful generation of USCs-iPSCs broadens the use of USCs for personalized cell therapy since USC-iPSCs have higher potency and self-renewal properties than USCs.

Our previous studies have shown the protective effects of flavonoids in various cancer cells [36,84,85,86]. Furthermore, 3,2’-DHF enhances the proliferation of mouse pluripotent stem cells and the pluripotency marker expression [41]. In our study, we found enhanced recovery of USCs and augmented proliferation of USCs prepared with 3,2′-DHF and 3,4′-DHF. Moreover, pretreatment with 3,2′-DHF and 3,4′-DHF led to enhance the EB formations and the differentiation into HPCs, and further differentiation into various hematopoietic lineage cells from USC-iPSCs. On the other hand, flavonoids treatment on EB did not affect the results of HPCs differentiation (data not shown). Given that differentiation capacity into HPCs of USC-iPSC is lower than that of PBMC-iPSCs, 3,2′-DHF and 3,4′-DHF treatment could be utilized to overcome the limitation of USC-iPSC’s differentiation capacity into HPCs. We also observed that flavonoid enhanced the population of CD34+/CD45+ cells in PBMC-iPSC [43]. Overall, these data suggest that flavonoids, 3,2′-DHF and 3,4′-DHF could possibly facilitate the efficient use of USCs by enhancing the efficiencies of USC isolation and differentiation of USC-iPSCs into HPCs and further into hematopoietic lineage cells.

## 5. Conclusions

USCs are promising sources of cells for cell therapy. We identified that Matrigel, Y-27632, and the flavonoids 3,2′-DHF and 3,4′-DHF improved the isolation efficiency, proliferation rate and differentiation potential of USCs. 3,2′-DHF and 3,4′-DHF also improved HPC generation from USC-iPSCs. These results could be applied to establish efficient preparation modalities of USCs and could potentially help establish strategies in regenerative medicine using USCs.

## Supplementary Materials

The following are available online at https://www.mdpi.com/2077-0383/9/3/827/s1, Figure S1: RNA sequencing of USCs, ADSCs, and WJ-MSCs. Figure S2: RNA sequencing of PBMCs, USCs, ESCs, USC-iPSCs, and PBMC-iPSCs. Figure S3: Chromosome abnormalities of USC-iPSCs. Figure S4: Flow cytometry analysis of HPC markers of differentiated USC-iPSC-2 derived HPCs. Table S1: Primer sequences.
