# Supplementary material for: Improved Isolation and Culture of Urine-Derived Stem Cells (USCs) and Enhanced Production of Immune Cells from the USC-Derived Induced Pluripotent Stem Cells
Source: J Clin Med. 2020 Mar 18;9(3):827. doi: 10.3390/jcm9030827 (PMC7141314; doi:10.3390/jcm9030827)
Supplement: Supplementary file 1 [file jcm-09-00827-s001.pdf]

Journal of
Clinical Medicine

MDPI

Supplementary Table 1. Primer sequences

Accession no.	Gene	Forward primer sequence (5'-3')	Reverse primer sequence (5'-3')	Application
NM_004360	E-cadherin	CAGCACGTACACAGCCCTAA	ACCCACCTCTAAGGCCATCT	epithelial markers
NM_021101	Claudin1	TTGACTCCTTGCTGAATCTGAG	TTCTGCACCTCATCGTCTTC	
NM_002538	Occludin	CTTCAGGCAGCCTCGTTACA	TCCTCCTCCAGCTCATCACA	
NM_003380	Vimentin	CTGCCTCTTCCAAACTTTTCC	GTGATGCTGAGAAGTTTCGTTG	fibroblast markers
NM_000474	Twist1	CTCAGCTACGCCTTCTCG	ACTGTCCATTTTCTCCTTCTCTG	
NM_001365518	Fibronectin	CTGGCCAGTCCTACAACCAG	CGGGAATCTTCTCTGTCAGC	
NM_006516	SLC2A1	CTTTTCTGTTGGGGGCATGAT	CCGCAGTACACACCGATGAT	renal epithelial markers
NM_000425	L1CAM	CAAGCCCGAAGTGCAGTTC	CTGGCAAAGCAGCGGTAGAT	
NM_000901	NR3C2	CACAGCACTGGTTCCTCAG	TTTGCCTGCTAAGCGGTTGA	
NM_002046.5	GAPDH	AATCCCATCACCATCTTCCAG	ATGACCCTTTTGGCTCCC	housekeeping gene
NM_004364.5	C/EBPα	CCACGCCTGTCCTTAGAAAG	CCCTCCACCTTCATGTAGAAC	adipogenic markers
NM_138712.4	PPARγ	GAGCCCAAGTTTGAGTTTGC	GCAGGTTGTCTTGAATGTCTTC	
NM_001442.3	FABP4	CATGTGCAGAAATGGGATGG	AACTTCAGTCCAGGTCAACG	
NM_001177800.2	Adiponectin	AAGGAGATCCAGGTCTTATTGG	ACCTTCAGCCCCGGGTAC	
NM_152860	Osterix	CTCCCTCTCCCTTTTCTCTCT	GGAGCCATAGTGAACTTCCTC	osteogenic markers
NM_001015051	Runx2	CGCCTCACAAACAACCACAG	TCACTGTGCTGAAGAGGCTG	
NM_004598	Osteonectin	ATGGGAATGAGTTGGCTGG	CCTCTTTGTCCTTTGGTCCC	
NM_000088	COL1A1	CCCCTGGAAAGAATGGAGATG	TCCAAACCACTGAAACCTCTG	osteogenic/ chondrogenic marker
NM_000493	COL10A1	ACGATACCAAATGCCCACAG	GTACCTTGCTCTCCTCTTACTG	chondrogenic markers
NM_000346	SOX9	ACTTGCACAACGCCGAG	CTGGTACTTGTAATCCGGGTG	
NM_001135	Aggrecan	TGTGGGACTGAAGTTCTTGG	AGCGAGTTGTCATGGTCTG	
NM_002701	OCT4	CAATTTGCCAAGCTCCTGAAG	GTTGCCTCTCACTCGGTTC	hPSC pluripotency markers
NM_003106	SOX2	TTCACATGTCCCAGCACTAC	TCCATGCTGTTTCTTACTCTC	
NM_024865	NANOG	CAGAAATACCTCAGCCTCCAG	GCCACCTCTTAGATTTCATTCTCTG	

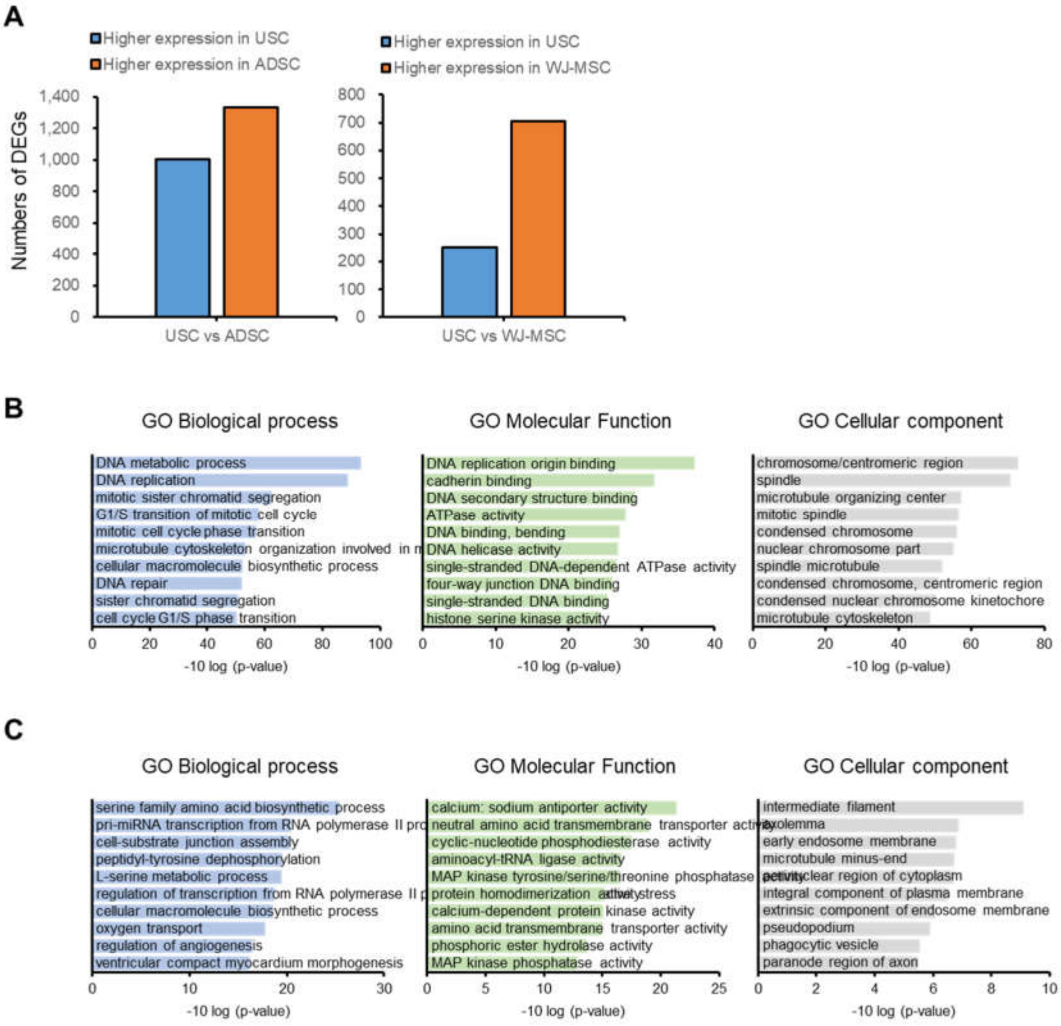

Supplementary Figure 1. RNA sequencing of USCs, ADSCs, and WJ-MSCs. (A) The number of differentially expressed genes between USCs and ADSCs, and between USCs and WJ-MSCs (q-value ≤0.01). (B) Functional enrichment analysis of more highly expressed genes in USCs (ADSC vs USC) and (C) Functional enrichment analysis of more highly expressed genes in USCs (WJ-MSC vs USC).

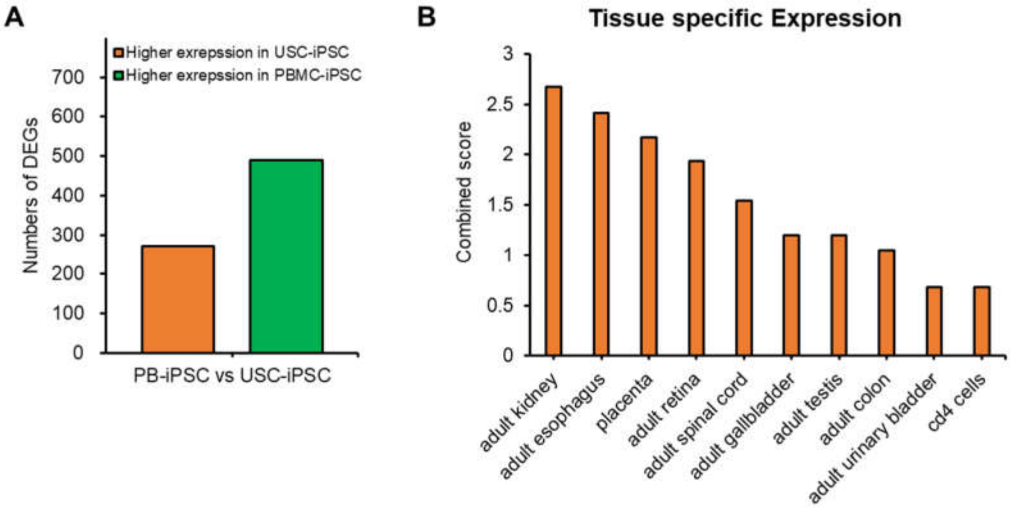

Supplementary Figure 2. RNA sequencing of PBMCs, USCs, ESCs, USC-iPSCs, and PBMC-iPSCs. (A) The differentially expressed genes between PBMC-iPSCs and USC-iPSCs are shown as upregulated genes (q-value ≤ 0.01). (B) Tissue-specific gene expression in USC-iPSCs compared with PBMC-iPSCs.

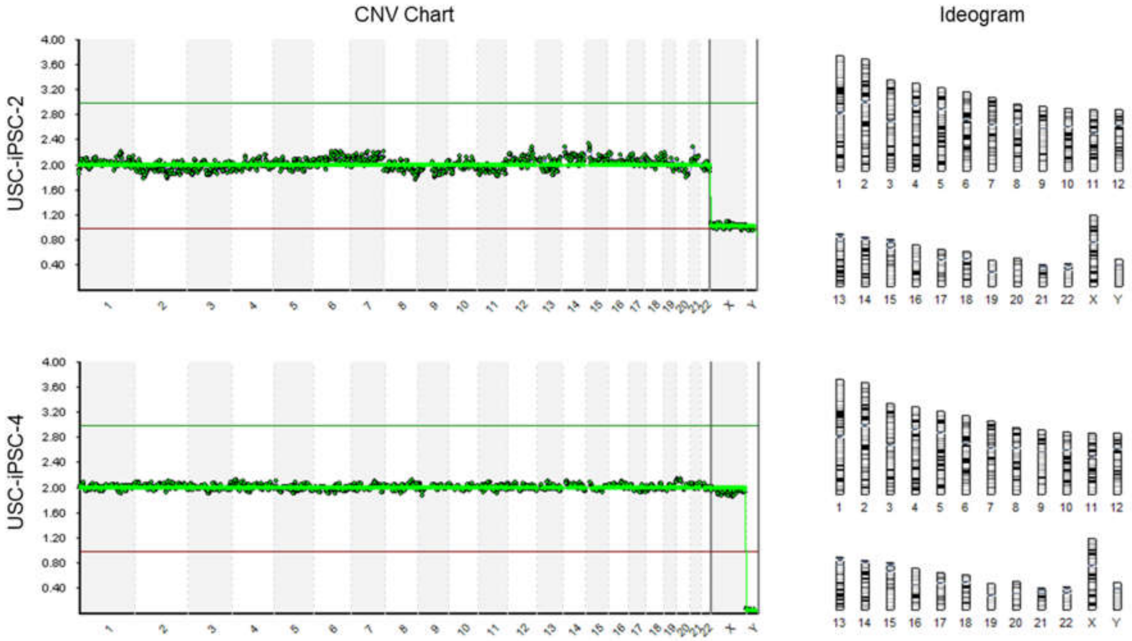

Supplementary Figure 3. Chromosome abnormalities of USC-iPSCs.

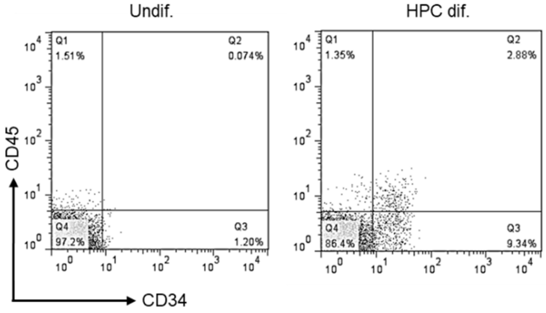

Supplementary Figure 4. Flow cytometry analysis of HPC markers of differentiated USC-iPSC-2 derived HPCs.
